# Evidence of a spatial auto‐correlation in the browsing level of four major European tree species

**DOI:** 10.1002/ece3.6577

**Published:** 2020-07-20

**Authors:** Robert Hagen, Rudi Suchant

**Affiliations:** ^1^ Forest Research Institute of Baden‐Württemberg (FVA) Freiburg Germany; ^2^ Leibniz Institute for Zoo and Wildlife Research Berlin Germany

**Keywords:** beech, fir, forest inventory, Morans'I, oak, roe deer, spruce

## Abstract

1. The contribution of spatial processes to the spatial patterns of ecological systems is widely recognized, but spatial patterns in the ecology of plant‐herbivore interactions have rarely been investigated quantitatively owing to limited budget and time associated with ecological research. Studies of the level of browsing on various tree species reported either no spatial auto‐correlation or a small effect size. Further, the effects of disturbance events, such as hurricanes, which create large forest openings on spatial patterns of herbivory are not well understood.
2. In this study, we used forest inventory data obtained from the federal state of Baden‐Württemberg (Southern Germany) between 2001 and 2009 (grid size: 100 × 200 m) and thus, after hurricane Lothar struck Southern Germany in 1999. We investigated whether the browsing level of trees (height ≤ 130 cm) in one location is independent of that of the neighborhood.
3. Our analyses of 1,758,622 saplings (187.632 sampling units) of oak (*Quercus*), fir (*Abies*), spruce (*Picea*), and beech (*Fagus*) revealed that the browsing level is characterized by a short distance spatial auto‐correlation.
4. The application of indicator variables based on browsed saplings should account for the spatial pattern as the latter may affect the results and therefore also the conclusions of the analysis.

## Graphical abstract

Spatial auto‐correlation for both regeneration density and browsing level is rather the rule than an exception. Accounting for spatial pattern in forestry will have important consequences for the design of forest inventories and management practices.

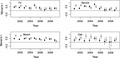

## 1 INTRODUCTION

Although tree recruitment in the northern Hemisphere has been impacted by large herbivores for thousands of years (Sommer, Fahlke, Schmölcke, Benecke, & Zachos, 2009), anthropogenic changes may be the most important recent factor affecting forest development (Tinner et al., 2013; Whitlock, Colombaroli, Conedera, & Tinner, 2017) and ungulate density (Bradshaw, Hannon, & Lister, 2003) as well as the relationship between them. It is widely recognized that the population dynamic of large herbivores is impacted by forest changes (Gaillard et al., 2003; Gill, Johnson, Francis, Hiscocks, & Peace, 1996). Research conducted over the last decade has highlighted, that large herbivores affect tree recruitment and species composition and thus future forest development as well (Bernard et al., 2017; Hidding, Tremblay, & Côté, 2013; Kuijper, Cromsigt, et al., 2010; Nuttle, Ristau, & Royo, 2014).

Browsing by large herbivores is frequently perceived as a major challenge for tree recruitment in Europe (Kupferschmid & Heiri, 2019), North America (Devaney, Pullen, Cook‐Patton, Burghardt, & Parker, 2020; Saucier, Champagne, Côté, & Tremblay, 2019), and Asia (Tamura & Yamane, 2017). However, forestry management remains largely disconnected from the management of large herbivore populations (Beguin, Tremblay, Thiffault, Pothier, & Côté, 2016; Reimoser, 2003). During the 18th century, forest management in Europe focussed on the consequences of anthropogenic changes for sustainable forestry practices, such as the loss of tree recruitment area due to industrial development and the intensive utilization of domestic animals (see Carlowitz, 1713). Until the early 20th century, the effects of large herbivores on tree recruitment and thus on forest development were largely ignored because large herbivores were of relatively low abundance, both in North America (Leopold, Bean, & Norman, 1943) and in Europe (Breitenmoser, 1998; Jędrzejewska, Jędrzejewski, Bunevich, Miłkowski, & Krasiński, 1997). During the 1940s and 1950s, however, awareness of the effects of large herbivores on tree recruitment in the Northern Hemisphere increased (Aldous, 1944; Leopold et al., 1943). Among the approaches developed to quantify the utilization of forest plants by large herbivores (Aldous, 1944; Reimoser, 2000) was that of Zai (1964), who in 1964 proposed a determination of the percentage of browsed trees (number of trees with browsed terminal buds divided by the total number of trees—the browsing level) as a robust index of roe deer (*Capreolus capreolus*) browsing. However, it was not until the 1980s that the browsing level of terminal buds was linked to tree growth and tree survival (Eiberle & Nigg, 1984, 1987). Thereafter, browsing‐level assessments gained increasing attention (Reimoser & Gossow, 1996; Welch, Staines, Scott, & French, 1992).Thus, the browsing‐level approach of Zai (1964), with its minimal required effort and observer independence, seemed to be a promising method to quantify the impact of large herbivores on tree regeneration (Morellet & Guibert, 1999). However, Reimoser, Odermatt, Roth, and Suchant (1997) and Senn and Häsler (2005) argued that a specific browsing level cannot be seen as direct damage caused by herbivores nor can it be related to a specific damage to forestry caused by herbivores. Reimoser (2003) pointed out that a higher browsing level might be a consequence of: (a) an increased need of large herbivores to engage in damaging activities, (b) an increase in the numbers of large herbivores, or (c) a change in forest structure resulting in the increased vulnerability of the saplings.

During the last two decades, numerous studies have contributed to a more holistic picture of the factors affecting the browsing of trees (Gerhardt, Arnold, Hackländer, & Hochbichler, 2013), including the population size of large herbivores (Beguin et al., 2016; Bernes et al., 2018; Chollet et al., 2016), forest management (Beguin, Pothier, & Prévost, 2009; Reimoser, Partl, Reimoser, & Vospernik, 2009), plant species composition (Boulanger et al., 2017; Mysterud, Askilsrud, Loe, & Veiberg, 2010; Nishizawa, Tatsumi, Kitagawa, & Mori, 2016), disturbance events (Royo, Collins, Adams, Kirschbaum, & Carson, 2010), landscape composition (Royo, Kramer, Miller, Nibbelink, & Stout, 2017), the combined effects of two large herbivores on plant communities (Faison, DeStefano, Foster, Motzkin, & Rapp, 2016) and those of season and herbivore density (Giroux, Dussault, Tremblay, & Côté, 2016). Thus, any indicator variable drawing on information from browsed trees should be characterized by a high spatial and temporal variability (Kuijper et al., 2009; Kuijper, Cromsigt, et al., 2010; Kuijper, Jedrzejewska, et al., 2010). Even though earlier studies of ecological processes recognized the importance of spatial processes (Moran, 1950; Sokal & Oden, 1978b), the quantification of spatial patterns in ecological research is rare (Dormann, 2007). Given that tree regeneration occurs patchily (Yokozawa, Kubota, & Hara, 1999) or in waves (Wiegand, Moloney, & Milton, 1998) and that large herbivores select habitat patches (Moser, Schütz, & Hindenlang, 2006; Widmer et al., 2004), spatial auto‐correlation in both the sapling density and browsing level should be the rule rather than the exception. Indeed, several authors have observed that the regeneration of oak and beech trees is spatially clumped (Götmark & Kiffer, 2014; Kunstler, Curt, & Lepart, 2004). Kunstler and coauthors (Kunstler et al., 2004) concluded that the spatial patterns of trees are mainly affected by seed dispersal and the spatial variability of germination. This nonrandom distribution in the environment will presumably affect the habitat utilization of large herbivores, which in part feed on saplings (Kuijper et al., 2009). The decision to browse a tree is part of a hierarchical decision‐making process that incorporates various factors at different spatial scales (cf. Champagne, Moore, Côté, & Tremblay, 2018). On an individual level, browsing intensity may be largely determined by the amount and quality of the forage (Shipley & Spalinger, 1995), including its species richness (Ohse, Seele, Holzwarth, & Wirth, 2017). This relation holds in particular for roe deer (*Capreolus capreolus*), as a selective feeder with a small rumen capacity (Hofmann, 1989).

In Baden‐Württemberg (Southern Germany) the browsing of trees is mainly attributable to roe deer because other species of the family *Cervidae* are restricted to relatively small areas (Hagen, Haydn, & Suchant, 2018; Hagen, Kühl, Kröschel, & Suchant, 2019). The home range of roe deer individuals varies between 20 and 60 ha (0.2–0.6 km^2^) depending on the region, season, and landscape composition (Lovari, Serrao, & Mori, 2017; Morellet et al., 2013; Richard, Said, Hamann, & Gaillard, 2014). Within home range roe deer select not only high‐quality forage patches but also high‐quality plants within those patches (Moser et al., 2006), and food quantity and quality will likely impact roe deer numbers (Gaillard et al., 2003; Gill et al., 1996). In the study of Gill and coauthors (Gill et al., 1996), roe deer density correlated negatively with total conifer cover with a time lag of 6 years. Thus, based on the ecology of plant‐herbivore interactions, it can be expected that the browsing of trees will be characterized by spatial patterns. However, there have been no, or at best few (cf. Champagne et al., 2018; Morellet & Guibert, 1999; Ohse et al., 2017) attempts to quantify the spatial characteristics of the browsing level. Furthermore, the results of those studies indicated either no auto‐correlation (Champagne et al., 2018; Morellet & Guibert, 1999) or only negligibly small effect sizes (Ohse et al., 2017).

In the present study, data from forest inventories (grid size of 100 × 200 m) conducted in the German federal state of Baden‐Württemberg (predator‐free system; only red fox (*Vulpes vulpes*), which kills roe deer juveniles, are common in Baden‐Württemberg [Kämmerle, Niekrenz, & Storch, 2019]) between 2001 and 2009 were analyzed. We tested the assumption that the species‐specific browsing level of saplings (height ≤ 130 cm) at one location is independent of the species‐specific browsing level of saplings at neighboring locations. The spatial independence not only of the browsing level but also of the number of saplings per sampling unit (sapling density) was determined by calculating Moran's *I* (Moran, 1950). The results presented for four major tree species (*Abies, Picea, Fagus, Quercus*) in Europe highlight the need to quantify spatial patterns in plant‐herbivore ecology research and practice.

## 2 DATA AND METHODS

**FIGURE 1:**
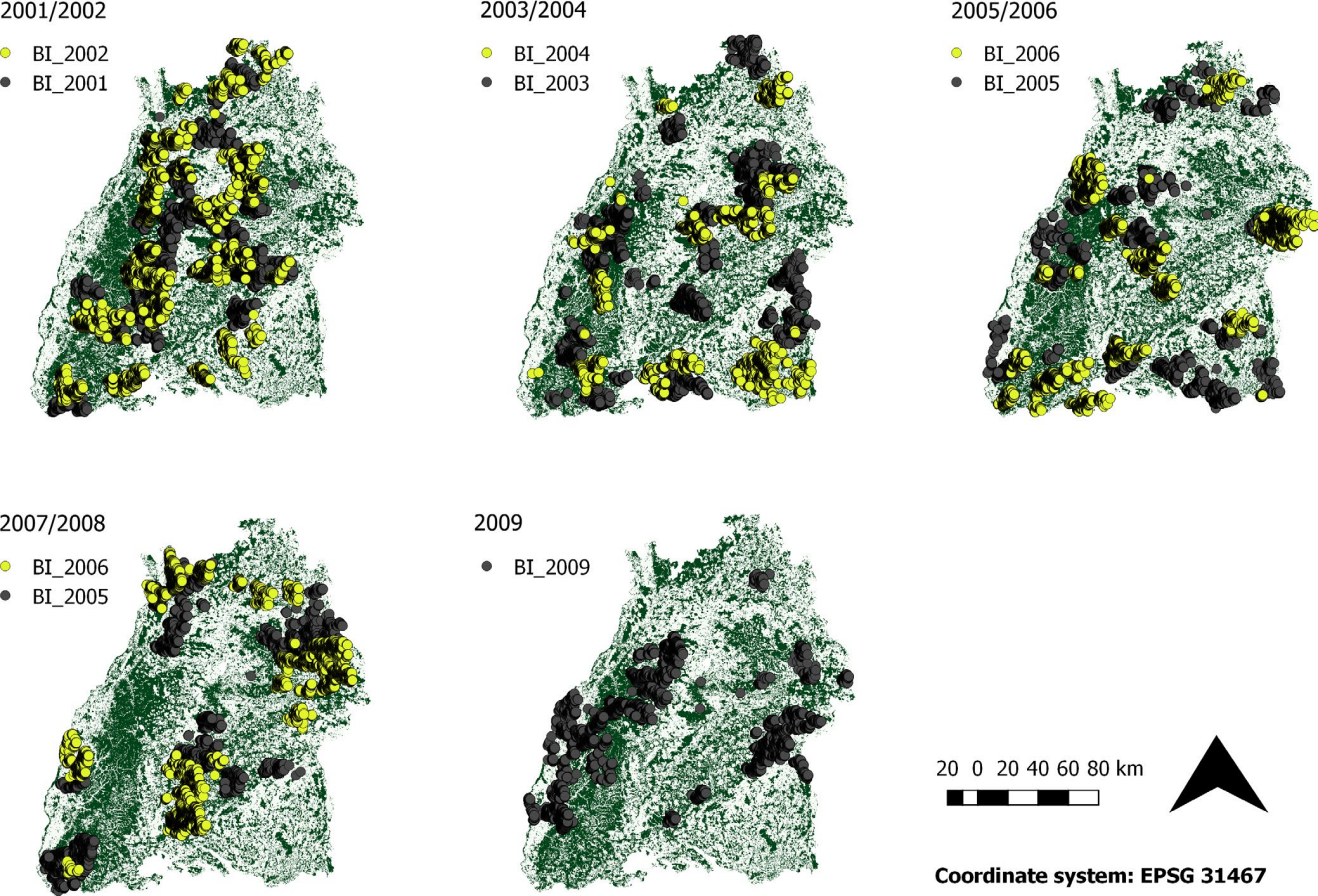
Sampling units of forest inventories (BI) in Baden‐Württemberg (2001–2009). The background shows the area covered by forest

**FIGURE 2:**
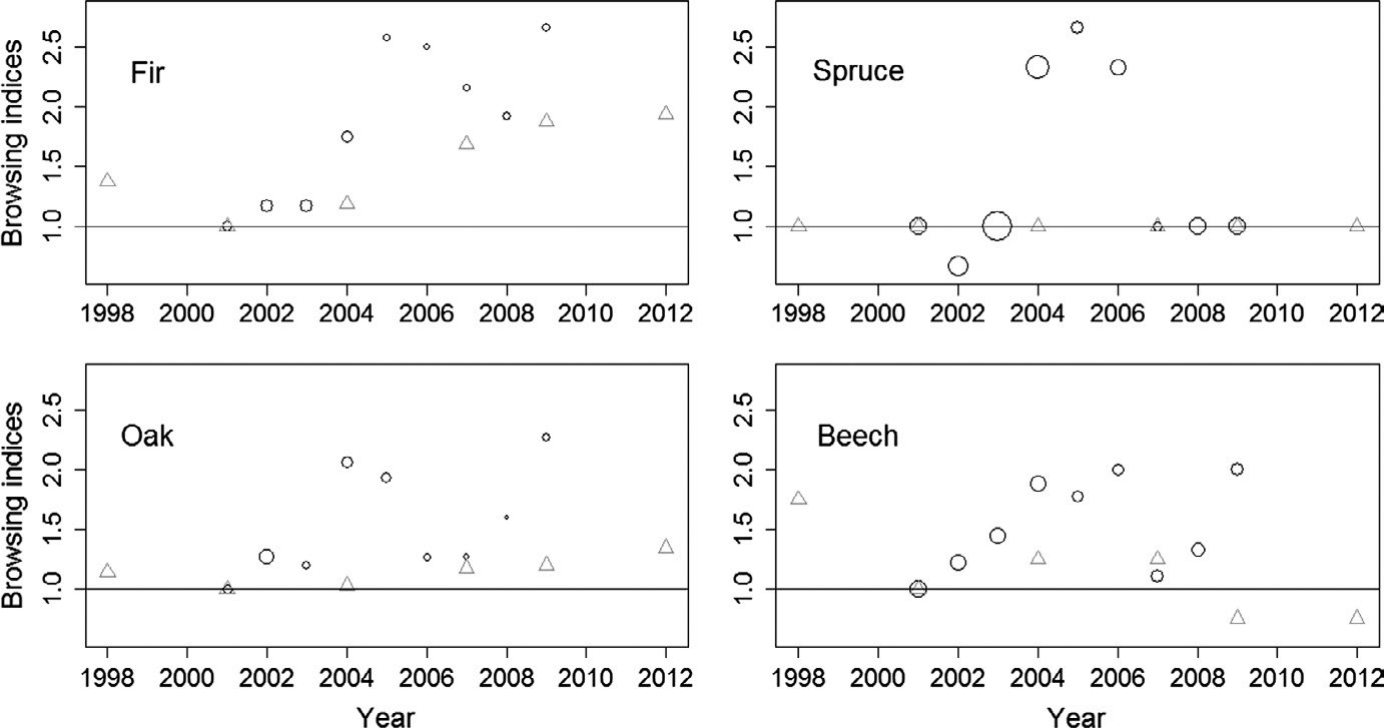
Temporal variation in the browsing of fir, spruce, beech, and oak. Open black circles show a browsing index based on data of forest inventories. To facilitate visual comparisons, all time series were normalized by the browsing level for 2001. The size of the circle represents the mean sapling density (the greater the circle the greater the mean number of saplings per sampling unit). Open gray triangles represent a browsing index derived from data of the Forstliches Gutachten Baden‐Württemberg—it represents the proportion of hunting grounds in Baden‐Württemberg that reported a high browsing level (>50%). To facilitate visual comparisons, time series were normalized by the value of 2001. We choose 2001 as a reference year as it represents a local minimum of the browsing level in Baden‐Württemberg

Since 1998, forest cultural undertakings (“Betriebe”) of the German federal state of Baden‐Württemberg has made use of forest inventory data to estimate timber production (Nothdurft, Borchers, Niggemeyer, Saborowksi, & Kändler, 2009). The inventory is conducted once per decade at the level of one “Betrieb” and collects data on the amount, age, and spatial distribution of tree species within a predefined grid (100 × 200 m, cf. Figure S1). The sampling units were marked by a steel pipe embedded in the ground in order to prevent visual detection. The collected information included the number of young trees (height ≤ 130 cm) within a sampling unit (circle with *r* = 1.5 m and an area of 7.1 m^2^) with and without browsing. A browsed tree was defined as a tree whose terminal bud was browsed during the last 3 years. Each sampling unit contained information describing a maximum of 90 saplings per tree species (density of 12.68 saplings per m^2^). In sampling units where the number of saplings exceeded this density, saplings deemed to be representative with respect to the height distribution of the regeneration and the overall browsing level were sampled. For this study, we analyzed data of four tree species, fir (*Abies*, number of saplings [*N*] = 238,471), Norway spruce (*Picea*, [*N*] = 715,120), beech (*Fagus*, [*N*] = 694,854), and oak (*Quercus*, [*N*] = 110,176) for the period 2001–2009 (Table 1). The annual sample covering several distinct regions in Baden‐Württemberg (cf. Figure 1) may or may not have appropriately represented the sapling density and the browsing level for the federal state of Baden‐Württemberg. We thus compared the browsing level determined in forest inventories with the results of the “Forstliches Gutachen Baden‐Württemberg” (cf. Figure 2), an official management tool to estimate both the browsing level and the possibility to reach forest management objectives. Since 1983, a survey has been conducted every third year for each hunting ground in Baden‐Württemberg (*N* ≈ 6,000, size of the hunting grounds ≈300–400 ha). In December 1999, hurricane Lothar struck Eastern France (Storms et al., 2006) and Southern Germany (Erb, Odenthal‐Kahabka, & Püttmann, 2004), creating large openings in the respective forests. In the federal state of Baden‐Württemberg, 30 million solid cubic meters were storm damaged (Erb et al., 2004). The resulting openings led to an increase in the number of saplings and thus to improved habitat quality for large herbivores in subsequent years (Storms et al., 2006; Widmer et al., 2004). Data of the “Forstliches Gutachten Baden‐Württemberg” suggested that the browsing intensity in Baden‐Württemberg declined to a local minimum in 2001. Thus, in this study, we used 2001 as the reference year (cf. Figure 2).

**TABLE 1:** Moran's I value for the browsing level and the regeneration density as well as basic information about the number of sample unit and the number of sampled trees

	2001	2002	2003	2004	2005	2006	2007	2008	2009
Fir									
Number of sampling units	3,526	6,930	7,728	2,522	2.637	2,922	2,441	2,437	4,550
Browsing level	0.12	0.14	0.14	0.21	0.31	0.3	0.26	0.23	0.32
Estimate I_100_	0.18	0.16	0.14	0.13	0.28	0.14	0.05	0.12	0.12
Standard deviation	0.03	0.02	0.02	0.04	0.03	0.04	0.05	0.05	0.03
*p* two sided	**<.0001**	**<.0001**	**<.0001**	**.0003**	**<.0001**	**.0002**	.23	.01	**<.0001**
Estimate I_200_	0.04	0.07	0.07	0.06	0.15	0.03	0	0.04	0.07
Standard deviation	0.04	0.03	0.03	0.05	0.05	0.06	0.07	0.07	0.04
*p* two sided	.31	.01	.009	.26	.001	.64	.93	.5	.04
Total number of saplings	24,136	56,776	64,729	18,613	12,490	12,524	11,870	12,928	24,405
Mean sapling density	6.85	8.19	8.34	7.38	4.74	4.29	4.86	5.34	5.36
Estimate I_100_	0.22	0.25	0.23	0.12	0.16	0.07	0.09	0.07	0.11
Standard deviation	0.03	0.02	0.02	0.04	0.03	0.04	0.05	0.05	0.03
*p* two sided	**<.0001**	**<.0001**	**<.0001**	.0006	**<.0001**	.06	.04	.12	**<.0001**
Estimate I_200_	0.1	0.12	0.09	0.1	0.1	0.03	0	0.02	0.04
Standard deviation	0.04	0.03	0.03	0.05	0.06	0.07	0.07	0.07	0.04
*p* two sided	.009	**<.0001**	.0008	.04	.03	.57	.93	.71	.26
Spruce									
Number of sampling units	6,114	9,176	11,890	5,169	3,414	5,469	3,319	5,074	4,727
Browsing level	0.03	0.02	0.03	0.07	0.08	0.07	0.03	0.03	0.03
Estimate I_100_	0.1	0.12	0.23	0.21	0.32	0.1	0.04	0.06	0.02
Standard deviation	0.02	0.02	0.01	0.02	0.03	0.03	0.04	0.03	0.03
*p* two sided	**<.0001**	**<.0001**	**<.0001**	**<.0001**	**<.0001**	**.0002**	.37	.05	.5
Estimate I_200_	0.04	0.02	0.1	0.12	0.2	0.03	0	0.01	0.01
Standard deviation	0.03	0.02	0.02	0.03	0.04	0.04	0.07	0.05	0.04
*p* two sided	.22	.43	**<.0001**	.0005	**<.0001**	.37	.93	.73	.87
Total number of saplings	67,897	122,154	231,414	80,737	28,392	55,564	20,990	56,642	51,330
Mean sapling density	11.01	13.31	19.46	15.62	8.31	10.16	6.32	11.16	10.86
Estimate I_100_	0.26	0.27	0.22	0.14	0.2	0.17	0.05	0.11	0.12
Standard deviation	0.02	0.02	0.01	0.02	0.03	0.03	0.04	0.03	0.03
*p* two sided	**<.0001**	**<.0001**	**<.0001**	**<.0001**	**<.0001**	**<.0001**	.29	.0004	**<.0001**
Estimate I_200_	0.16	0.16	0.14	0.14	0.13	0.11	0.03	0.06	0.07
Standard deviation	0.03	0.02	0.02	0.03	0.04	0.04	0.07	0.05	0.04
*p* two sided	**<.0001**	**<.0001**	**<.0001**	.03	.002	.003	.64	.22	.08
Beech									
Number of sampling units	10,256	11,804	9,827	5,648	5,649	9,525	8,274	7,654	5,888
Browsing level	0.09	0.11	0.13	0.17	0.16	0.18	0.1	0.12	0.18
Estimate I_100_	0.19	0.15	0.19	0.17	0.21	0.17	0.11	0.17	0.11
Standard deviation	0.02	0.01	0.02	0.02	0.02	0.02	0.02	0.02	0.03
*p* two sided	**<.0001**	**<.0001**	**<.0001**	**<.0001**	**<.0001**	**<.0001**	**<.0001**	**<.0001**	**<.0001**
Estimate I_200_	0.09	0.06	0.06	0.08	0.11	0.08	0.05	0.08	0.03
Standard deviation	0.02	0.02	0.03	0.03	0.03	0.03	0.03	0.03	0.04
*p* two sided	**<.0001**	.005	.009	.01	**.0001**	.003	.04	.003	.35
Total number of saplings	114,331	119,092	102,478	57,008	41,352	75,272	66,482	68,950	49,889
Mean sapling density	11.15	10.09	10.43	10.09	7.32	7.9	8.03	9.01	8.47
Estimate I_100_	0.34	0.34	0.21	0.21	0.17	0.13	0.23	0.17	0.06
Standard deviation	0.02	0.01	0.02	0.02	0.02	0.02	0.02	0.02	0.03
*p* two sided	**<.0001**	**<.0001**	**<.0001**	**<.0001**	**<.0001**	**<.0001**	**<.0001**	**<.0001**	.02
Estimate I_200_	0.23	0.19	0.11	0.12	0.08	0.05	0.11	0.12	0.01
Standard deviation	0.02	0.02	0.03	0.03	0.03	0.03	0.03	0.03	0.04
*p* two sided	**<.0001**	**<.0001**	**<.0001**	**.0002**	.003	.03	**<.0001**	**<.0001**	.66
Oak									
Number of sampling units	2,155	3,005	1,977	1,858	1,,895	2,022	1,521	1,079	1,902
Browsing level	0.15	0.19	0.18	0.31	0.29	0.19	0.19	0.24	0.34
Estimate I_100_	0.11	0.14	0.08	0.1	0.2	0.1	0.04	0.01	0.1
Standard deviation	0.05	0.04	0.06	0.05	0.04	0.05	0.08	0.14	0.05
*p* two sided	.02	**.0002**	.16	.05	**<.0001**	.04	.6	.87	.04
Estimate I_200_	0.03	0.04	0.02	0.05	0.09	0.03	0.01	0.01	0.05
Standard deviation	0.08	0.06	0.1	0.08	0.06	0.08	0.14	0.37	0.08
*p* two sided	.71	.46	.82	.54	.14	.64	.88	.81	.48
Total number of saplings	13,865	28,316	11,343	14,165	13,475	10,218	6,050	2,815	9,929
Mean sapling density	6.43	9.42	5.74	7.62	7.11	5.05	3.98	2.61	5.22
Estimate I_100_	0.21	0.23	0.32	0.29	0.19	0.3	0.12	0.03	0.17
Standard deviation	0.05	0.04	0.05	0.05	0.04	0.05	0.07	0.11	0.05
*p* two sided	**<.0001**	**<.0001**	**<.0001**	**<.0001**	**<.0001**	**<.0001**	.11	.72	.0004
Estimate I_200_	0.07	0.1	0.17	0.13	0.14	0.12	0.04	0.00	0.15
Standard deviation	0.08	0.06	0.1	0.08	0.06	0.08	0.13	0.00	0.08
*p* two sided	.37	.12	.09	.12	.02	.1	.67	.99	.05

Note

Bold values highlight a significant spatial auto‐correlation under the global *p*‐value of .05.

We calculated Moran's *I* for a predefined neighborhood *d*_nb_ (*d*_nb_ *= *100 m, 200 m,…, 500 m that cover an area of 0.03 km^2^, 0.126 km^2^,…,0.785 km^2^) covering mean values of home range size published for roe deer to test for spatial independence:

(1) $$I=\left(N/W\right)*\left(\left(\sum_{i}\left(\sum_{j}w_{\mathrm{ij}}\left(x_{i}‐x_{\text{mean}}\right)\left(x_{j}‐x_{\text{mean}}\right)\right)\right)/\sum_{i}{\left(x_{i}‐x_{\text{mean}}\right)}^{2}\right)$$

where *N* is the number of spatial units, *x* the browsing level, *x*_mean_ the mean browsing level, *w_ij_* the weight according to the defined neighborhood (*w_ij_ = *0 for *i = j*; *w_ij_ = *0 for *d*(*i*, *j*) > *d*_nb_) and *W* the sum of all *w_ij_*.

Thus, *I* was calculated as the correlation coefficient for pairs of points considered as neighbors.

A calculated value of *I* significantly less or greater than 0 negated the hypothesis that the browsing of young trees (height ≤ 130 cm) is a spatially independent process. The Bonferroni correction was used to correct for multiple testing effects. Statistical calculations were carried out using R version 3.4.4 (R Core Team 2018) and the R package spdep (Bivand et al., 2006).

## 3 RESULTS

**FIGURE 3:**
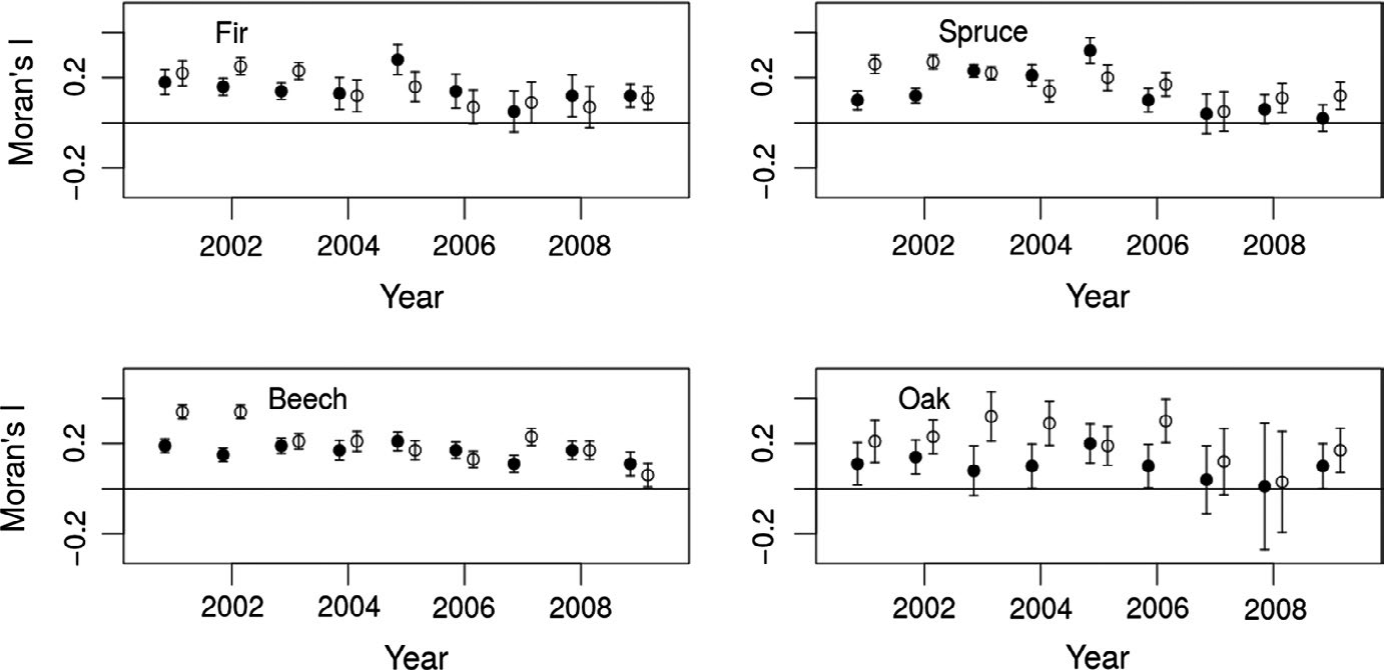
Moran's I for the browsing level (filled circles) and the sapling density (open circles) based on a neighborhood distance of 100 m. The bars cover twice the square root of the estimated variance

Between 2001 and 2009, the browsing level of the four studied tree species (fir, spruce, beech, and oak) varied considerably, by a factor of 2 (Table 1 and Figure 2). Compared to the reference year 2001, high browsing levels were determined for all tree species during the period 2004–2006 (Figure 2). Sapling density peaked during 2001–2003 (cf. Table 1 and Figure 3—local maximum for oak in 2002, beech in 2001, spruce and fir in 2003). The estimates for *I* as well as the estimated *p*‐values showed strong inter‐annual variations for both the browsing level and the sapling density (Table 1, Figures 3 and S4). Both the browsing level and the sapling density were characterized by a positive spatial auto‐correlation (Figures 3 and S4). Thus, Moran's *I* (browsing level) was greatest for a neighborhood distance (*d*_nb_) of 100 m (Table 1, Figure 3) and decreased for an increasing *d*_nb_ up to 500 m (Figures S2 and S3 show Moran's *I* for *d*_nb_ up to 500 m) indicating that both variables were characterized by a short‐distance auto‐correlation. Although the parameter estimates for *I* did not exceed 0.32 (browsing level) and 0.34 (sapling density), the data did not support the hypothesis of spatial independence for either sapling density (*d*_nb_ = 100 m, all tree species) or browsing level (*d*_nb_ = 100 m, fir, spruce, and beech). The maximal values for *I* (browsing level) were calculated for the year 2005 and independently of the tree species (Figures 3 and S4) and corresponded to a rather high overall browsing level (Figure 2). Thus, the local maximum of both, the browsing level and the parameter estimate of Moran's *I* lagged 5–6 years behind hurricane Lothar. Maximal values for *I* (sapling density) were calculated for fir, spruce and beech during the years 2001 and 2002 (directly after Lothar created large openings) and for oak during the period 2003–2006 (Figures 3 and S4).

## 4 DISCUSSION

In this study, Moran's *I* (Moran, 1950) was calculated to test for the spatial independence of (a) the sapling density of four tree species (fir, spruce, beech, and oak ≤ 130 cm) and (b) the browsing level of those trees, using forest inventory data for Baden‐Württemberg (2001–2009). The results showed an auto‐correlation of the two variables for a distance of 100 m (and partly for larger neighborhood distances up to 300 m; cf. Figures S2 and S4).

The unambiguous demonstration of a spatial auto‐correlation for sapling density and browsing level was initially surprising. Although the ecology of plant‐herbivore interactions predicts the existence of spatial auto‐correlation in sapling density and in the browsing level of trees, previous research reported either the absence of spatial auto‐correlation in the browsing level (Champagne et al., 2018; Morellet & Guibert, 1999) or rather small values of Moran's *I* (Ohse et al., 2017). This mismatch between ecological prediction and recent findings might reflect the uncertainty in decision‐making processes owing to incomplete information (Hagen, Kramer‐Schadt, Fahse, & Heurich, 2014). The limited budget and time invested in ecological research have led to a rather limited spatial‐temporal resolution of the available data sets and thus in a low power to detect spatial auto‐correlations. For example, previous sampling units frequently corresponded to a single year (Morellet & Guibert, 1999), two years (Champagne et al., 2018) or three years (Ohse et al., 2017) and were sampled in relatively small study areas (<6 km^2^ Morellet & Guibert, 1999, Champagne et al., 2018; 75 km^2^ Ohse et al., 2017). Our inventory data for the year 2007 provide an example of how incomplete information can result in a failure to detect spatial auto‐correlations (Table 1). The estimated value of Morans *I* (year 2007) supports the hypothesis of spatial independence for both sapling density and browsing level. However, while this conclusion might be true for the sampled year/region, it does not imply that data on sapling density and browsing level can generally be regarded as spatially independent variables (Table 1, Figures 2 and S4). Differences in Moran's *I* between tree species may be due to the different dispersal strategies of the trees (Dormann, 2007; Yokozawa et al., 1999). The decrease in Moran's *I* (sapling density) for fir, spruce and beech throughout the period 2001–2009 likely reflected ecological processes initiated by hurricane Lothar in 1999. Only Moran's *I* (sapling density) for oak showed intensive year to year variations. Moran's *I* (browsing level) reached peak values in 2005, which coincided with the high values for the browsing level (cf. Figures 2 and 3). The difference between the maximum and minimum annual browsing levels of oak, fir, spruce, and beech was 20%, 19%, 6%, and 9%, respectively (Table 1). These differences together with Moran's *I* (browsing level) clearly show that browsing is a highly variable process both in time and in space. While this is well‐known in plant‐herbivore ecology (Beguin et al., 2016; Bernes et al., 2018; Sinclair & Krebs, 2002; Sokal & Oden, 1978a), our study is the first to show evidence that the browsing level of four major European tree species (fir, spruce, oak, and beech) is characterized by a significant short‐distance auto‐correlation. The fact that Moran's *I* of the browsing level and sapling density was more likely to be significant for a neighborhood distance of 100 m suggests that processes responsible for this spatial pattern were themselves characterized a by short‐distance spatial autocorrelation (Sokal & Oden, 1978b). The observed spatial pattern can be explained by four different responses (Sokal & Oden, 1978b): (1) to an environmental gradient (Model I): (2) to habitat patches that are heterogeneous among themselves but internally homogenous (Model II); (3) to the isolation caused by distance (Model III); and (4) to differences in historical factors (Model IV).

We suggest that the observed auto‐correlation of the sapling density is best explained by a combination of Model II, III, and IV. As for the observed spatial auto‐correlation of the browsing level, our results favor a combination of Model I, II, and III. Although forest practices are one major factor impacting the distribution of tree species and species composition in Germany (Model IV) hurricane Lothar created large openings in Baden‐Württemberg. These openings were homogenous (Model II) but separated from each other (Model III). The openings led to an increase in the overall sapling density between 2001 and 2003 (cf. Table 1 and Figure 2). High values were determined for Moran's *I* for the sapling density of oaks between 2003 and 2006, when the sampling units were characterized by relatively low to medium sapling densities (Table 1, Figure 2). Moran's *I* for the sapling densities of fir, spruce and beech were greatest for the years 2001 and 2002 and thus for years in which sapling density was highest (Models II and III). The high sapling density between 2001 and 2003 together with the inability of hunters to access hunting grounds in 2000 and 2001 (cf. Gaillard et al., 2003 for France) may have affected the population dynamic of roe deer in Baden‐Württemberg and in turn the overall browsing level (Model I). With respect to the results of Gill and coauthors, this might have affected the browsing of trees around the year 2005 (6 years after Lothar created the openings). In fact, not only the browsing level of oak, spruce, fir and beech (Figure 2) but also Moran's *I* of the browsing level (Figures 3 and S4) peaked during 2004–2006. These results suggest that hurricane Lothar initiated the following cascade: The storm‐damaged forest led to both an inability of hunters to access hunting grounds and an increase in the number of saplings → an increase in habitat suitability together with a decrease in hunting‐related mortality → an increase in roe deer numbers in subsequent years → and higher browsing levels. Thus, the spatial auto‐correlation of the browsing level for relatively short neighborhood distances (area of 0.03 km^2^ [Figure 3], 0.13 km^2^ [Figure S4] and 0.28 km^2^ [Figure S2] for spruce in 2005) might reflect not only the sapling density but also the selection process of roe deer within their home range, as the home range size varies between 0.2 and 0.6 km^2^ (Lovari et al., 2017; Morellet et al., 2013; Richard et al., 2014) and is smaller in forest areas (Lovari et al., 2017). If this was the case, then the analysis of datasets of browsed and unbrowsed trees using a grid size of 50 m or 25 m would be informative. Although definitively identifying the drivers of the spatial auto‐correlation in both the regeneration and the browsing level will be challenging, our findings highlight the importance of accounting for spatial patterns in plant‐herbivore ecology. In addition, the application of indicator variables based on browsed trees (cf. Chevrier et al., 2012; Maublanc, Bideau, Launay, Monthuir, & Gerard, 2016; Morellet et al., 2007; Pierson & De Calesta, 2015) should account for the spatial pattern in sapling density. It should also be noted that although forest inventories in Austria, Germany, and Switzerland are conducted using a grid size of 100 × 200 m or 100 × 100 m (Kupferschmid, 2018; Nothdurft et al., 2009; Ohse et al., 2017), the distance between the sample units used to obtain information on browsed trees is frequently <100 m (Ammer, 1996; Moser et al., 2006; Kuijper et al., 2009; Champagne et al., 2018) or ≤200 m (Heinze et al., 2011; Heuze, Schnitzler, & Klein, 2005; Kuijper, Jedrzejewska, et al., 2010; Morellet & Boscardin, 2001; Ohse et al., 2017; Partl, Szinovatz, Reimoser, & Schweiger‐Adler, 2002).

Thus, we suggest that every study using data on browsed trees should first investigate the existence and strength of spatial auto‐correlation. If the variable of interest is used as a target variable for any regression model or correlation analysis, then appropriate statistical methods should be applied (cf. Dormann et al., 2007). Otherwise, the assumption of independence of most standard statistical procedures will be violated and type I and II error rates might increase (Dormann et al., 2007; Legendre, 1993). Our study can be understood as a first step in a systematic investigation of short‐distance spatial autocorrelation phenomena in plant‐herbivore ecology. The insights obtained from those investigations will likely have important consequences for the design of forest inventories and the management practices derived from their results.

## CONFLICT OF INTEREST

Rudi Suchant and Robert Hagen disclosed no conflict of interests.

## AUTHOR CONTRIBUTION

**Robert Hagen:** Conceptualization (lead); Formal analysis (lead); Methodology (lead); Validation (equal); Visualization (lead); Writing‐original draft (lead); Writing‐review & editing (lead). **Rudi Suchant:** Conceptualization (supporting); Data curation (lead); Formal analysis (supporting); Investigation (supporting); Methodology (supporting); Project administration (lead); Supervision (lead); Validation (equal); Writing‐original draft (supporting); Writing‐review & editing (supporting).

## Supporting information

Figures S1‐S4

## Data Availability

Data visualized in Figures 2, 3 and S2–S4 were archived in Dryad (https://doi.org/10.5061/dryad.4xgxd256m). Data of forest inventories (raw data shown in Figure 1) will not be made publicly available as data contain sensitive information (human subject data in time and space) about timber production in the federal state of Baden‐Württemberg.
