# Supplementary material for: Evidence of a spatial auto‐correlation in the browsing level of four major European tree species
Source: Ecol Evol. 2020 Jul 20;10(15):8517–27. doi: 10.1002/ece3.6577 (PMC7417255; doi:10.1002/ece3.6577)
Supplement: Supplementary file 1 — Figures S1‐S4 [file ECE3-10-8517-s001.docx]

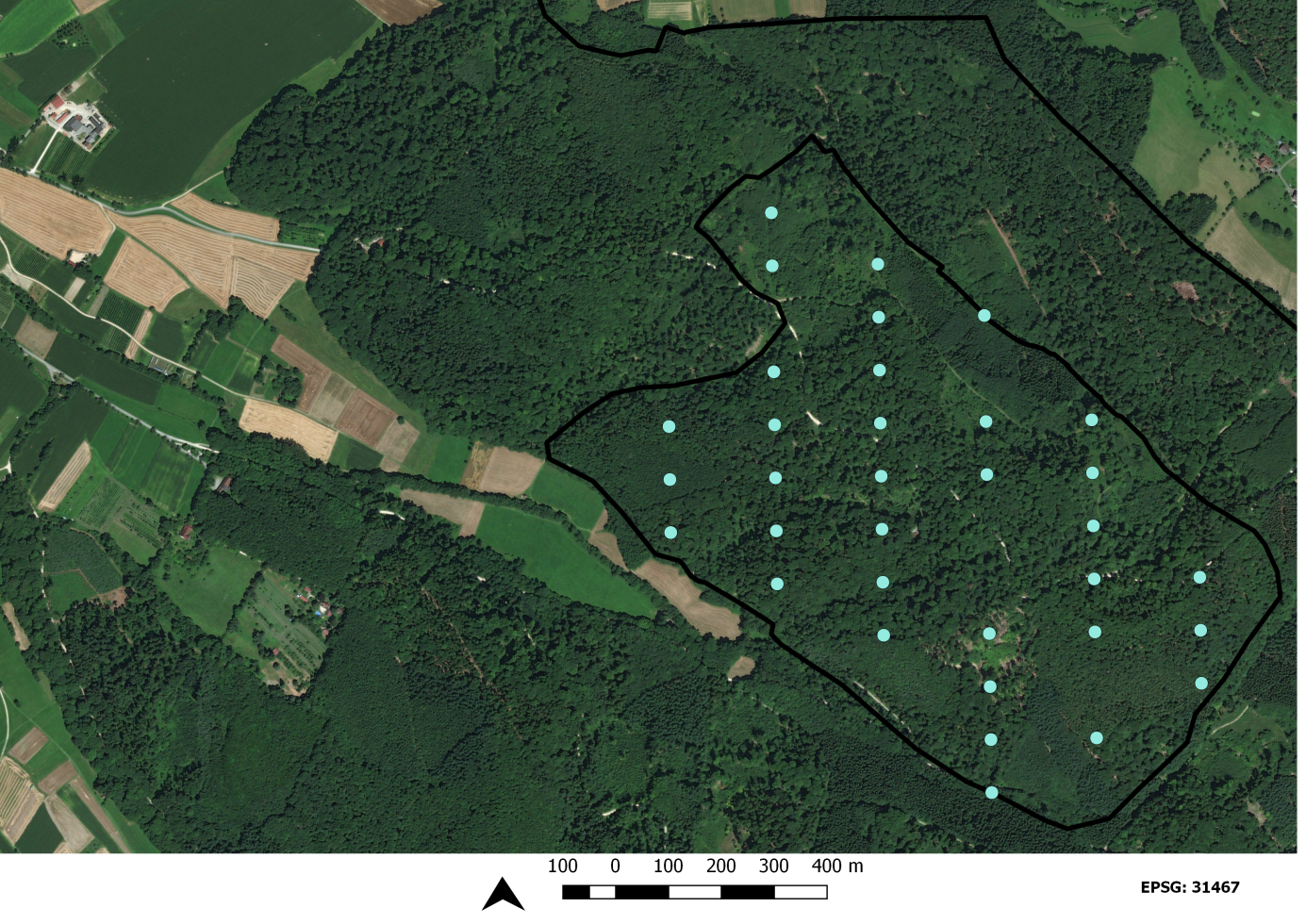


Fig. S1 Example of BI-sampling units (filled circles) in a forest patch (a hunting ground of the Forstliches Gutachten Baden-Württemberg) according to the predefined grid of 100x200m. Each sampling unit equals a circle with r =1.5 m and thus, an area of 7.1 m². Please note, only sampling units that lie in forest patches and not include parts of roads or pads were matter of interest for forest inventories.

| 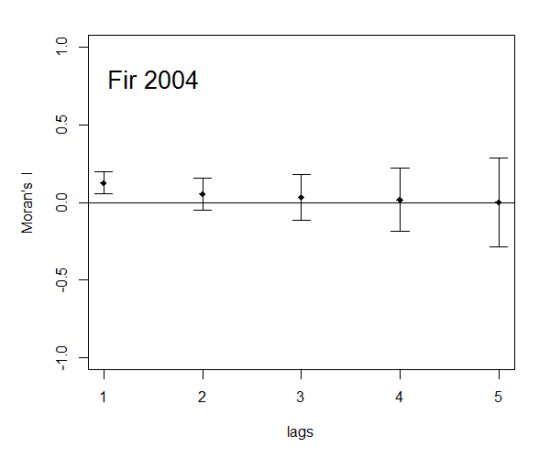 | 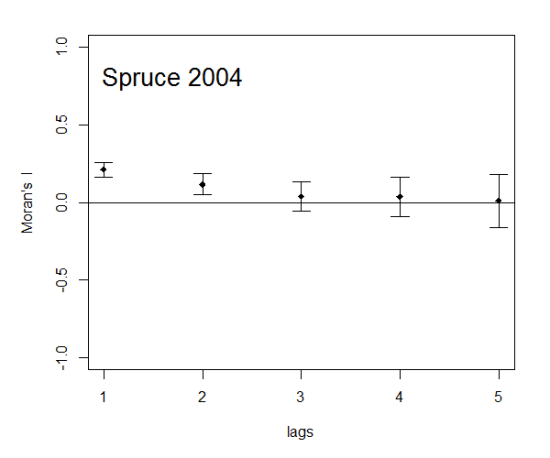 |
| --- | --- |
| 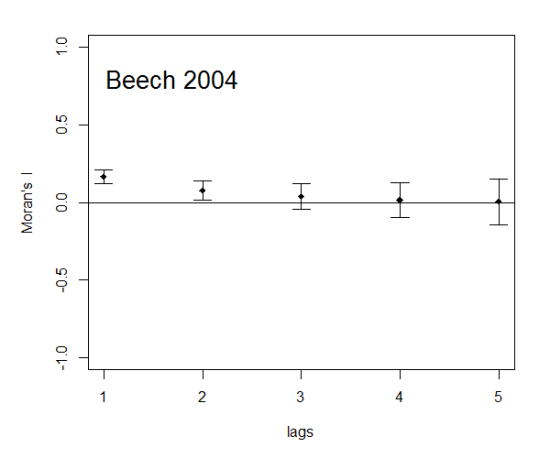 | 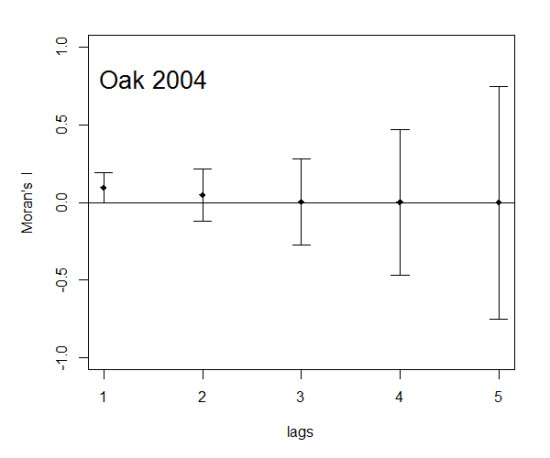 |

Fig. S2 Moran’s *I* for the browsing level of fir, spruce, beech and oak in 2004. A lag-length of 1 corresponds to a distance of 100 m. The bars cover twice the square root of the estimated variance.

| 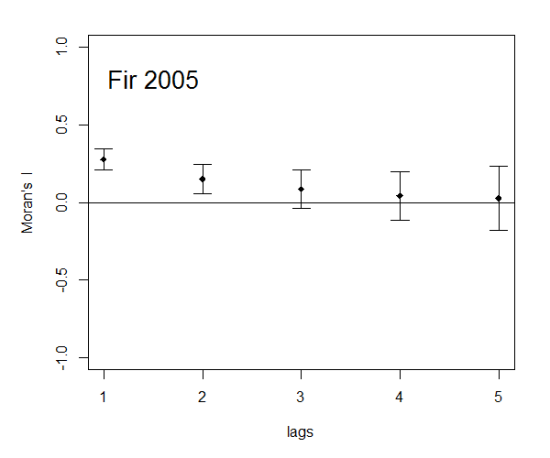 | 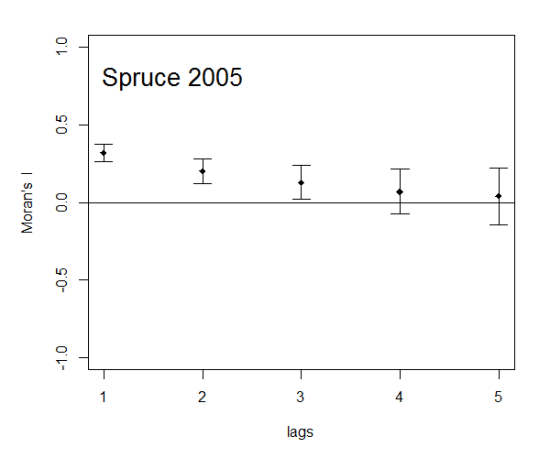 |
| --- | --- |
| 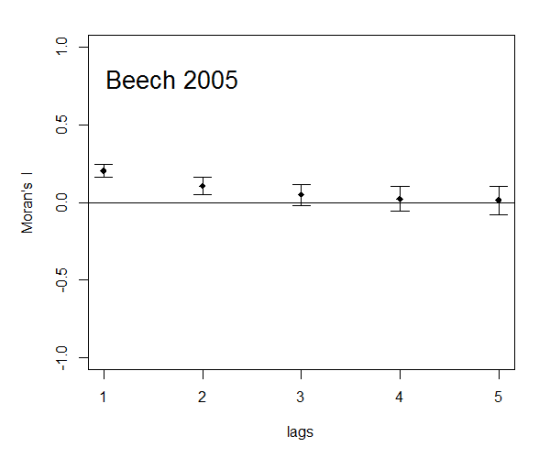 | 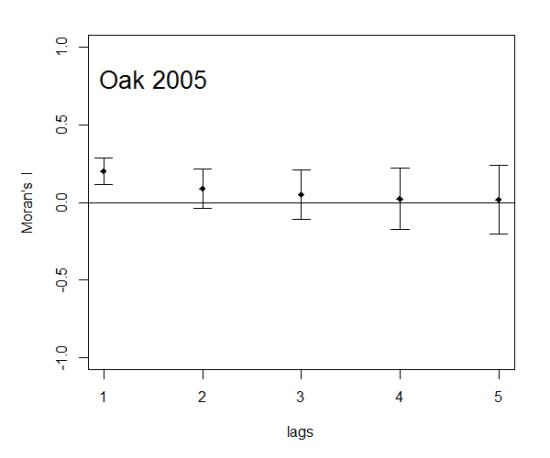 |

Fig. S3 Moran’s *I* for the browsing level of fir, spruce, beech and oak in 2005. A lag-length of 1 corresponds to a distance of 100 m. The bars cover twice the square root of the estimated variance.


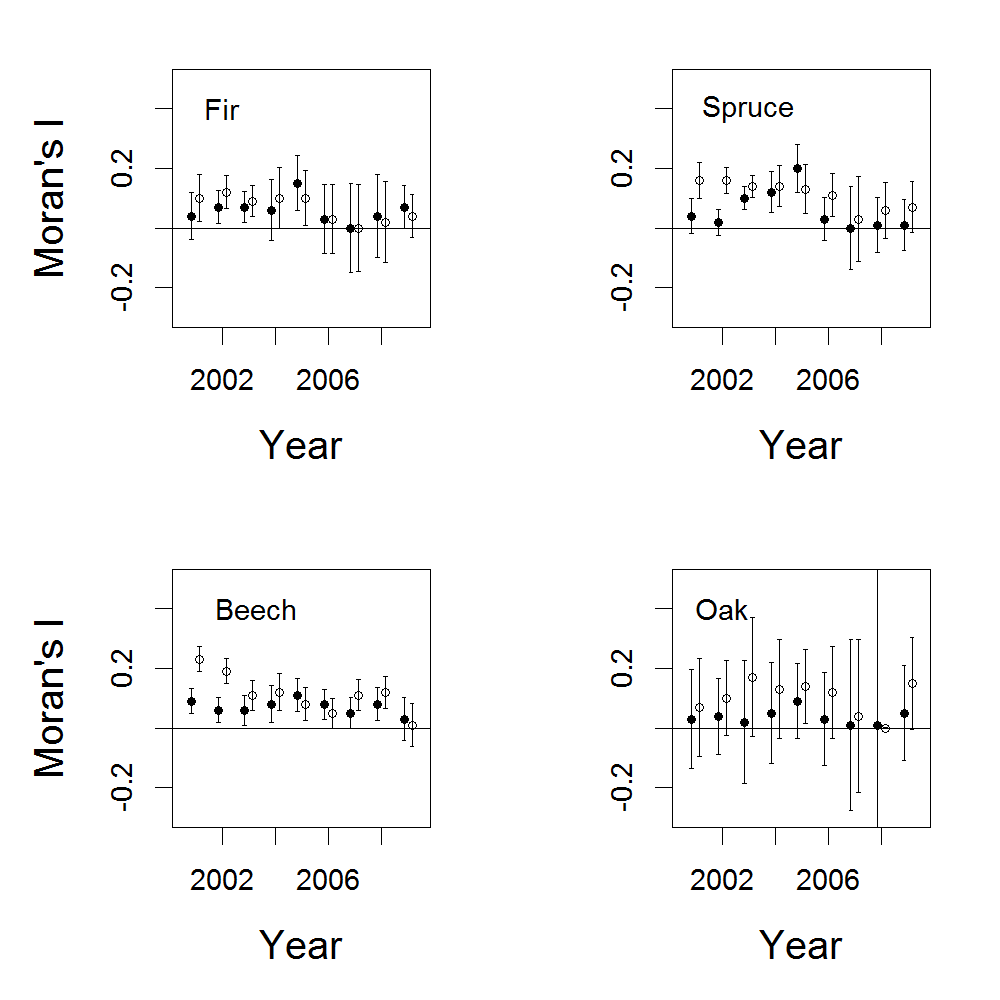
 Fig. S4 Moran’s *I* for the browsing level (filled circles) and the regeneration density (open circles) based on a neighbourhood distance of 200 m. The bars cover twice the square root of the estimated variance.
